# Feasibility and diagnostics of the Frontal Assessment Battery (FAB) in amyotrophic lateral sclerosis

**DOI:** 10.1007/s10072-022-06438-5

**Published:** 2022-10-06

**Authors:** Edoardo Nicolò Aiello, Federica Solca, Silvia Torre, Laura Carelli, Roberta Ferrucci, Alberto Priori, Federico Verde, Nicola Ticozzi, Vincenzo Silani, Barbara Poletti

**Affiliations:** 1 IRCCS Istituto Auxologico Italiano, Department of Neurology and Laboratory of Neuroscience, Piazzale Brescia 20, Milano, 20149 Italy; 2 PhD Program in Neuroscience, School of Medicine and Surgery, University of Milano-Bicocca, Monza, Italy; 3 Aldo Ravelli Center for Neurotechnology and Experimental Brain Therapeutics, Department of Health Sciences, International Medical School, University of Milan, Milan, Italy; 4 ASST Santi Paolo E Carlo, San Paolo University Hospital, Milan, Italy; 5 IRCCS Ca’ Granda Foundation Maggiore Policlinico Hospital, Milan, Italy; 6 Department of Pathophysiology and Transplantation, “Dino Ferrari Center”, Università Degli Studi di Milano, Milan, Italy

**Keywords:** Frontal assessment battery, Amyotrophic lateral sclerosis, Cognitive screening, Executive, Diagnostics, Psychometrics

## Abstract

**Background:**

The present study aimed at evaluating the diagnostic properties of the Frontal Assessment Battery (FAB) in non-demented ALS patients by addressing the Edinburgh Cognitive Behavioural ALS Screen (ECAS) as the gold standard, as well as by examining the association between its administrability and scores with motor-functional measures.

**Materials:**

*N* = 348 consecutive patients were administered the ECAS and FAB. Disease severity (ALSFRS-R), duration, progression rate (ΔFS), and stages (via King’s and Milano-Torino systems) were considered. Administrability rates and prevalence of below-cut-off FAB scores were compared across clinical stages; regression models allowed to test whether, net of the ECAS-Total, motor features predicted the probability of the FAB not being administrable and of a defective FAB score. Intrinsic and post-test diagnostics were explored against a combined defective ECAS-Executive and ECAS-Fluency scores.

**Results:**

85.3% of patients managed to complete the FAB. FAB administrability rates decreased with advanced clinical stages, whereas the prevalence of below-cut-off FAB scores did not. The probability of the FAB not being administrable was predicted only by lower ALSFRS-R-bulbar and ALSFRS-R-upper-limb scores; no motor features, but the ECAS-Total, predicted a below-cut-off performance on the FAB. Raw and adjusted FAB scores showed high accuracy (AUC = .85 and .81, respectively) and good intrinsic and post-test properties.

**Discussion:**

The FAB is featured by optimal diagnostics for detecting executive deficits in ALS, provided that it can be administered according to its original, standardized procedure, and thus that patients have sufficiently spared motor abilities to complete the test.

## Background

In ALS patients, the feasibility of the Frontal Assessment Battery (FAB) [1] as a screener for deficits of executive functioning (EF)—which are highly prevalent/incident in this population [2]—has been historically questioned due to its heavy reliance on motor-/verbal-mediated responses, and thus, the possibility of upper-limb disabilities/dysarthric features undermining test execution and/or confounding test scores [3].

Notwithstanding that disease-specific cognitive screeners [4] undisputedly come with the highest level of recommendation for use in both clinical practice [5] and research [6] as addressed to ALS patients, the FAB still appears to be a rather widespread test to screen for EF deficits in this population [7], being also supported by seemingly sound clinimetric evidence [8].

However, available information on the diagnostics of the FAB in ALS patients has the intrinsic downfall of coming from studies that compared it against gold standard measures that were disease-nonspecific [9, 10]. Analogously, those reports that focused on its feasibility in this population, albeit to the noble aim of accommodating motor disabilities, included off-label adjustments to the administration/scoring procedure [11, 12]—which should be avoided, as making test results incomparable to the original norms and clinimetrics that were pursuant to a standardized protocol [13]. Furthermore, despite having been proposed that the FAB is mostly suitable for ALS patients in the early stages—i.e., as long as they have sufficiently spared articulatory and upper-limb functioning, this reasonable hypothesis has been to this day tested by merely relating FAB scores to motor measures or disease duration [10–12, 14–16], with not all studies agreeing on the expected association between lower FAB scores with more severe/advanced disease [10, 12, 14, 16]. Most importantly, this last finding would not allow per se to conclude that the FAB is not suitable for patients in advanced stages, as it might simply reflect the fact that cognitive decline goes along with disease progression in ALS [17]—something that has also yielded when adopting the Edinburgh Cognitive and Behavioural ALS Screen (ECAS) [18], notwithstanding that it controls for motor disabilities [19].

Given the above premises, the present study aimed at (1) evaluating the diagnostic properties of the original FAB in a large, clinic-based cohort of non-demented ALS patients by addressing the ECAS as the gold standard, as well as at (2) examining in-depth the association between its administrability and scores with motor-functional measures.

## Methods

### Participants

*N* = 348 consecutive, non-demented ALS patients referred to the IRCCS Istituto Auxologico Italiano between 2017 and 2021 were recruited. Exclusion criteria were (1) (ALS unrelated) neurological/psychiatric diagnoses, (2) severe general-medical conditions, and (3) uncorrected hearing/vision deficits. This study was approved by the Ethics Committee of IRCCS Istituto Auxologico Italiano (I.D.: 2013_06_25); the participants provided informed consent and the data were treated according to current regulations.

### Materials

All patients underwent the Italian ECAS [20] and FAB [21]. Disease severity was assessed via the ALS Functional Rating Scale-Revised (ALSFRS-R) [22], whereas progression rate (ΔFS) was computed as follows: 48—ALSFRS-R)/disease duration in months [23]. Disease staging was retrieved via both King’s [24] and Milano-Torino systems [25].

### Statistics

The administrability rate and prevalence of below-cut-off FAB scores [21] across King’s and Milano-Torino clinical stages were compared by means of *χ*^2^ tests of independence.

Moreover, two logistic regressions were run to test whether, net of global cognition (i.e., the ECAS-Total), motor features (i.e., ALSFRS-R-bulbar, ALSFRS-R-respiratory, ALSFRS-R-upper-limb, and ALSFRS-R-lower-limb scores, disease duration, and ΔFS) predicted the probability of (1) the FAB being administrable or not and (2) a below- vs. above-cut-off score on the FAB. Age, education, and sex were covaried within the first model, whereas only sex in the second one—since the FAB cutoff is adjusted for age and education [21]. In such models, Bonferroni’s correction was applied when selecting significant predictors (*α*_adjusted_ = 0.05/number of target predictors, i.e., excluding covariates).

The association between FAB and ECAS scores was tested via Bonferroni-corrected Spearman’s correlations (since FAB scores did not distribute normally—i.e., skewness and kurtosis values ≥|1| and |3|, respectively [26]).

FAB diagnostics were explored via receiver-operating characteristics (ROC) analyses by addressing as the gold standard a combination of a below-cut-off performance on the ECAS-Executive and on the ECAS-Fluency subscales [20], which operationalized EF deficits. Within such an analysis, sensitivity, specificity, positive, and negative predictive values (PPV; NPV) and likelihood ratios (LR + ; LR −) were computed at the optimal cutoff identified via Youden’s *J* statistic. By postulating that up to 50% of patients could present with EF deficits operationalized as above [2], the minimum sample size was estimated, for a single-test ROC analysis [27], at *N* = 82 (allocation ratio of 1, i.e., *N* = 41 patients with vs*. N* = 41 without EF deficits), by addressing an AUC = 0.7, *α* = 0.05 and 1-*β* = 0.95.

The significance level was set at *α* = 0.05; missing values were excluded pairwise. Analyses were run via R 4.1.0 (https://cran.r-project.org/).

## Results

**Fig. 1:**
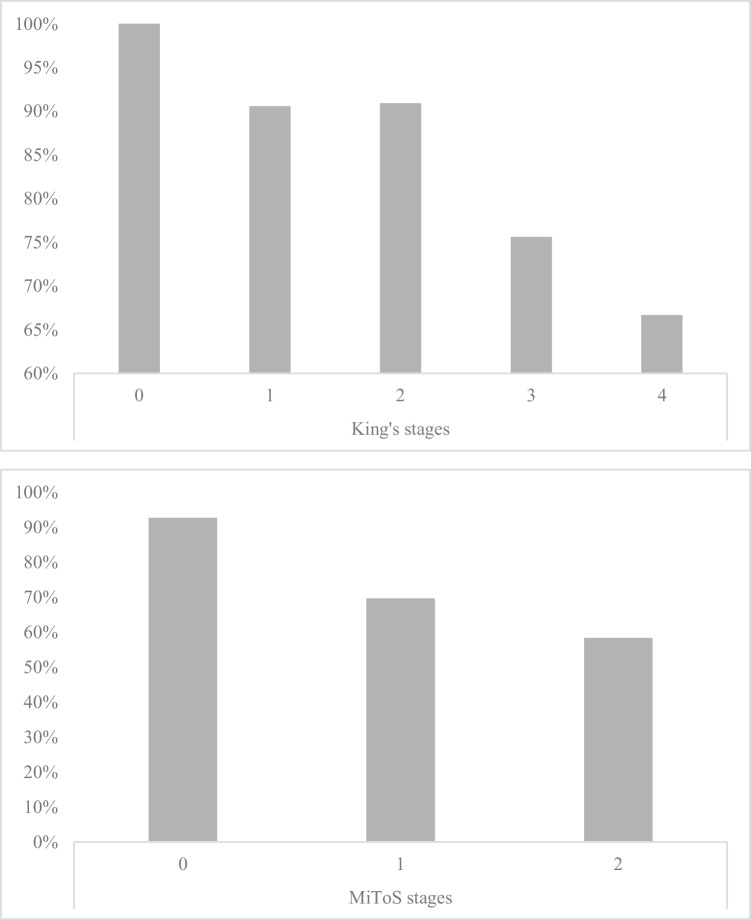
FAB administrability rates across King’s (upper panel) and MiToS stages (lower panel)

Table 1 shows the background and clinical measures of patients that managed to complete the FAB (297/348, i.e., 85.3% of the whole cohort). When addressing the whole cohort (i.e., *N* = 348 patients) FAB administrability rates decreased with advanced both King’s (*χ*^2^(4) = 16.98; *p* = 0.002) and Milano-Torino stages (*χ*^2^(2) = 32.33; *p* < 0.001) (Fig. 1). By contrast, the prevalence of below-cut-off FAB scores did not vary as a function of either King’s (*χ*^2^(4) = 2.89; *p* = 0.576) or Milano-Torino stages (*χ*^2^(2) = 3.6231; *p* = 0.164).

**Table 1:** Background and clinical features of patients that underwent the FAB

***N***	297
Age (years)	63.1 ± 11.2 (28–88)
Sex (M/F)	62.3%/37.7%
Education (years)	11.6 ± 4.4 (5–24)
Handedness (right/left)	94.6%/5.4%
Disease duration (months)	17.4 ± 15.9 (2–120)
ALSFRS-R	
Total	39.2 ± 5.5 (23–48)
Bulbar	10.5 ± 2 (4–12)
Spinal–lower limbs	11.2 ± 3.8 (0–16)
Spinal–upper limbs	6.2 ± 1.8 (1–8)
Respiratory	11.3 ± 1.5 (3–12)
ΔFS	0.8 ± 0.8 (0–5.3)
KSS	
Stage 0	1.9%
Stage 1	36.2%
Stage 2	34%
Stage 3	23.4%
Stage 4	4.5%
MiToS	
Stage 0	76.6%
Stage 1	20.8%
Stage 2	2.6%
PEG	0.3%
NIV	4.1%
Genetics	
* C9orf72*	7.1%
* SOD1*	2.7%
* TARDBP*	3.4%
* FUS*	0.3%
FAB	
Raw scores	15.7 ± 2.1 (8–18)
Below-cut-off scores^a^	12.1%
ECAS	
Total	99.8 ± 18.6 (31–129)
ALS-specific	73.8 ± 15.1 (21–97)
ALS-nonspecific	26 ± 5 (9–34)
Language	23.4 ± 3.9 (10–28)
Fluency	16.3 ± 5.6 (0–24)
Executive	34 ± 7.8 (7–47)
Memory	14.6 ± 4.6 (1–22)
Visuo-spatial	11.4 ± 1 (6–12)
ECAS-CI	0.7 ± 0.9 (0–5)

**Notes.** *ΔFS* progression rate, *ALS* amyotrophic lateral sclerosis, *ALSFRS-R* Amyotrophic Lateral Sclerosis Functional Rating Scale-Revised, *ECAS* Edinburgh Cognitive and Behavioural ALS Screen, *F* female, *FAB* Frontal Assesment Battery, *KSS* King’s staging system, *M* male, *MiToS* Milano-Torino staging system, *NIV* non-invasive ventilation, *PEG* percutaneous endoscopic gastrostomy ^a^Appollonio et al. [21]

At *α*_adjusted_ = 0.007, the probability of the FAB not being administrable was predicted only by lower ALSFRS-R-bulbar (*b* =  − 0.42; *z* = -5.06; *p* < 0.001) and -upper-limb scores (*b* =  − 0.48; *z* =  − 4.45; *p* < 0.001). By contrast, in patients to whom the FAB could be administered, no motor features predicted, at *α*_adjusted_ = 0.007, a below-cut-off performance on the FAB. Within such models, the ECAS-Total did not yield significance in the former (*p* = 0.937), while being the only, negative predictor in the latter (*b* =  − 0.08; *z* =  − 6.12; *p* < 0.001).

At *α*_adjusted_ = 0.01, FAB scores were related to all ECAS subscales (0.33 ≤ *r*_*s*_(297) ≤ 0.65; *p* < 0.001), with the strongest correlations being however found with ECAS-Executive (*r*_*s*_(297) = 0.64) and ECAS-Fluency subscores (*r*_*s*_(297) = 0.65).

Thirty-three out of 297 patients that managed to complete the FAB were classified as executively impaired based on a combination of a below-cut-off score on the ECAS-Executive and ECAS–Fluency subscales (11.1%).

FAB raw scores yielded, at an optimal cutoff of 15 ≤ (*J* = 0.5), high accuracy in discriminating executively impaired vs. executively unimpaired patients (AUC = 0.85; *SE* = 0.03; CI 95% [0.78, 0.91]), with an optimal balance between sensitivity (78.8%) and specificity (71.6%), a low PPV (25.7%) in the face of a high NPV (96.4%) and adequate likelihood ratios (LR +  = 2.77; LR −  = 0.3). According to such a cutoff, 34% of patients were classified as impaired on the FAB. Age- and education-adjusted scores [21] on the FAB were similarly accurate (AUC = 0.81; *SE* = 0.04; CI 95% [0.73, 0.88]) and featured, at the optimal cut-off (15.6 <; *J *= 0.5), by overall comparable diagnostics (sensitivity = 81.8%; specificity = 68.6%; PPV = 24.5%; NPV = 96.8%; LR+ = 2.6; LR- = 0.27). According to this latter cutoff derived on adjusted scores, 37% of patients were classified as impaired.

## Discussion

The present study provides relevant insight into the feasibility and diagnostics of the FAB in ALS patients. For the first time, it has been herewith shown that the FAB is less/not administrable to patients in the advanced stages of the disease, as well as that, when administrable, it does not relate to disease duration/severity. Indeed, the FAB administrability rates decreased with advanced King’s/MiToS stages, but the prevalence of defective scores on it did not. Moreover, net of cognitive status, bulbar and upper-limb deficits predicted the non-administrability of the FAB. By contrast, such motor features were not predictive of a below-cut-off FAB score—which was instead predicted only by global cognitive levels.

This finding suggests that previously found associations between lower FAB scores and a more severe/longer disease [11, 15] are likely to be spurious, i.e., mediated by a decline in cognitive functioning—which is known to go along with disease progression [17, 19]. By contrast, as reasonably expectable, dysarthric features and upper-limb impairments do impact on the administrability of the screener—which, indeed, heavily relies on verbal- and motor-mediated responses. However, it appears that the previously hypothesized proportion of patients that could not be administered the FAB, i.e., up to 50% [7], is an overestimation—since, herewith, such an estimate yielded to be of 15.7%. Thus, modifications to the standardized administration/scoring procedure should be avoided, and the screener thus applied only to suitable patients. In support of such a suggestion, Osborne et al. [12] found no differences in FAB scores from ALS patients who received or not, based on their motor status, modified instructions.

As to FAB diagnostics, the present results are overall in line with a previous report by Barulli et al. [10], who similarly administered the original FAB to ALS patients with sufficiently spared motor abilities. The FAB indeed herewith proved to be highly accurate, with an optimal sensitivity–specificity balance, as well as featured by overall good post-test properties—except for a low PPV value, which however could be biased by the lower-than-expected prevalence of executively-impaired patients [28]. 

Notably, the FAB also strongly converged with ECAS-Executive and ECAS-Fluency scores, and this further supports the notion that it is a valid screener for EF deficits in this population.

In conclusion, the FAB is featured by optimal diagnostic properties for detecting EF deficits in ALS patients, provided that it can be administered according to its original, standardized procedure and thus patients have sufficiently spared articulatory and upper-limb functions to complete the test. Hence, although ALS-specific screeners (e.g., the ECAS) [18] still remain the gold-standard option for the cognitive/behavioral assessment in ALS patients, the FAB would feature itself as a valuable alternative to screen for EF impairment in this population within non-specialist clinical settings that may be less familiar with disease-specific tools [18, 29] and/or lack expertise in their administration/scoring procedures (e.g., general outpatient/inpatient services and neurology units or memory clinics). Indeed, it has to be noted that the FAB does not control for verbal-motor limitations and is not exhaustive in detecting the multi-dimensional nature of cognitive/behavioral changes characterizing ALS patients [22]. Nevertheless, the data herewith presented supports the adoption of the FAB as an EF measure within extensive cognitive evaluations addressed to ALS patients, as well as within retrospective studies that aim to analyze the data collected before the availability of ALS-specific screeners [18, 29].

Future studies are nevertheless needed to focus on a number of aspects that were not addressed within this study. Firstly, the contribution of disease duration/severity to the administrability of the FAB, as well as to its scores, should be assessed at the subtest level—i.e., linguistically mediated EF, motor-mediated EF, and inhibition, according to a recently proposed classification [30]. Secondly, longitudinal studies are needed to confirm the present, cross-sectional findings. Finally, it is advisable that the diagnostics and feasibility of the FAB be explored by stratifying ALS patients according to Strong et al.’s [2] criteria.

## Data Availability

Datasets associated with the present study are available upon reasonable request of interested researchers.
